# Four Dimensions of the Cardiac Myocyte Epigenome: from Fetal to Adult Heart

**DOI:** 10.1007/s11886-020-01280-7

**Published:** 2020-03-19

**Authors:** Carolin Rommel, Lutz Hein

**Affiliations:** 1 Institute of Experimental and Clinical Pharmacology and Toxicology, Faculty of Medicine, University of Freiburg, Albertstr. 25, 79104 Freiburg, Germany; 2 BIOSS Centre for Biological Signalling Studies, University of Freiburg, Freiburg, Germany

**Keywords:** Epigenetics, Atrial cardiac myocytes, Ventricular cardiac myocytes, Transcription factor, Transcriptome, DNA methylation

## Abstract

**Purpose of Review:**

Development, physiological growth and the response of the heart to injury are accompanied by changes of the transcriptome and epigenome of cardiac myocytes. Recently, cell sorting and next generation sequencing techniques have been applied to determine cardiac myocyte-specific transcriptional and epigenetic mechanisms. This review provides a comprehensive overview of studies analysing the transcriptome and epigenome of cardiac myocytes in mouse and human hearts during development, physiological growth and disease.

**Recent Findings:**

Adult cardiac myocytes express > 12,600 genes, and their expression levels correlate positively with active histone marks and inversely with gene body DNA methylation. DNA methylation accompanied the perinatal switch in sarcomere or metabolic isoform gene expression in cardiac myocytes, but remained rather stable in heart disease. DNA methylation and histone marks identified > 100,000 cis-regulatory regions in the cardiac myocyte epigenome with a dynamic spectrum of transcription factor binding sites. The ETS-related transcription factor ETV1 was identified as an atrial-specific element involved in the pathogenesis of atrial fibrillation.

**Summary:**

Thus, dynamic development of the atrial vs. ventricular cardiac myocyte epigenome provides a basis to identify location and time-dependent mechanisms of epigenetic control to shape pathological gene expression during heart disease. Identifying the four dimensions of the cardiac myocyte epigenome, atrial vs. ventricular location, time during development and growth, and disease-specific signals, may ultimately lead to new treatment strategies for heart disease.

## Introduction

The heart is the first organ to develop prenatally and continuously contracts throughout the entire life [1]. Multiple transcription factor networks control formation of the cardiac chambers during embryonic development [2•, 3, 4]. Cardiac myocytes from the first and second heart field form distinct areas of the four cardiac chambers and the conduction system of the mature heart [4, 5]. In the adult heart, cardiac myocytes are highly specialized and can be separated into myocytes of the conduction system, including pacemakers and rapid conducting cells, and working cardiac myocytes of the atria and ventricles. These types of cardiac myocytes also differ in their transcriptomes as revealed by single-cell RNA sequencing [6–8].

Due to early differentiation and specialization in their function, cardiac myocytes withdraw from cell cycle around the time of birth with very low rates of postnatal cell proliferation. In the adult human heart, the annual rate of cardiac myocyte proliferation is estimated to be below 1% [9, 10••]. Thus, in contrast to many other tissues in the body, the heart has a limited capacity to compensate for loss or damage of cardiac myocytes [11]. Thus, cardiac myocytes surviving an injury frequently react with cellular hypertrophy, altered sarcomere isoform expression, changes in mitochondrial metabolism, electrical remodelling and other functional and structural changes. Many of these events are based on or accompanied by altered gene expression regulated by transcriptional and posttranscriptional mechanisms [4, 12].

**Fig. 1:**
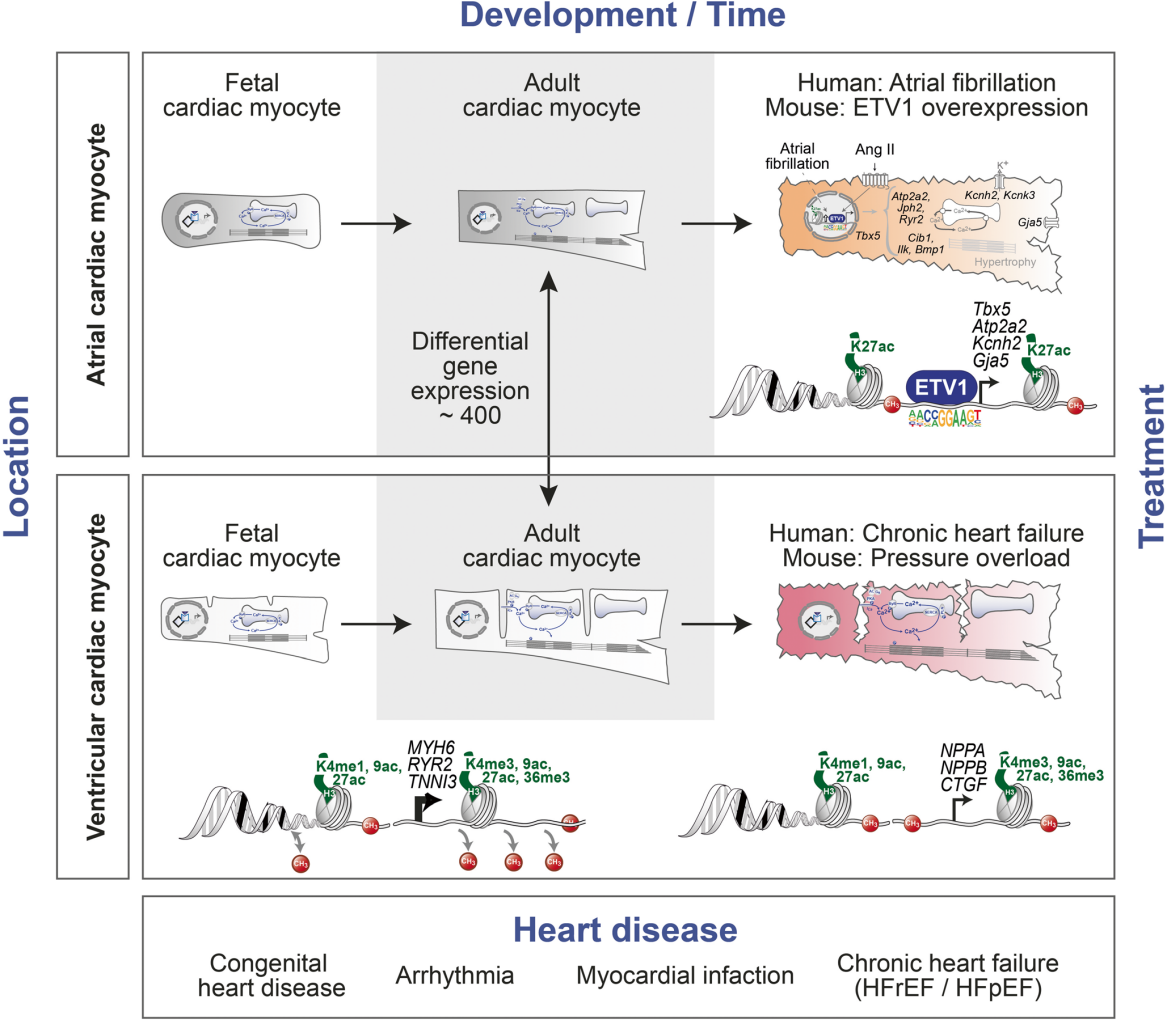
Four dimensions of the cardiac myocyte epigenome—from fetal to adult heart. Atrial cardiac myocytes (upper panel) show significantly different gene expression patterns compared with ventricular cardiac myocytes (lower panel) (grey background). Chromatin accessibility assessed by ATAC-seq in atrial cardiac myocytes combined with H3K27ac signature and RNA expression identified an ETV1-dependent gene regulatory network involved in atrial remodelling (upper panel). Epigenetic analysis during ventricular cardiac myocyte development showed that mCpG and canonical histone marks contribute to induce or repress cardiac myocyte genes. Induction of disease-associated genes in failing cardiac myocytes is accompanied by active histone modifications and no changes in gene body mCpG (lower panel)

Development, growth and the response to injury of cardiac myocytes are controlled by transcription factors, which orchestrate cardiac myocyte gene expression in close interaction with multiple layers of epigenetic regulation [13•, 14••, 15–21]. Essential epigenetic mechanisms modulating physiological and pathological gene expression include chromatin remodelling, histone modifications, DNA methylation and non-coding RNAs [22]. These factors control the cardiac myocyte transcriptome in a well-coordinated manner during development and in disease. Recent studies have mapped the dynamics of the mouse and human cardiac myocyte transcriptome and epigenome during prenatal development, postnatal maturation and growth and in chronic heart failure (Fig. 1) [13•, 14••, 23••, 24, 25].

## Isolation of Cardiac Myocyte Nuclei for Epigenetic Analysis

Epigenetic mechanisms are highly cell-type-specific processes and thus require isolation of distinct cell types (or nuclei) from cardiac tissue for precise analyses [26]. Thus, to uncover cardiac myocyte-specific epigenetic mechanisms, different methods were developed and tested to isolate cardiac myocytes or nuclei from heart tissue of different species. While isolation of intact cardiac myocytes from frozen tissue has remained quite challenging, the identification of myocyte-specific antigens in the nuclear membrane has led to a break-through. Initial studies used cardiac troponin I or T for isolation of cardiac myocyte nuclei [9] until the centrosome protein PCM1 (pericentriolar material 1) was found to accumulate specifically at the outer nuclear membrane of cardiac myocytes but not in non-myocytes of the heart [10••]. Antibodies recognizing PCM1 have been successfully used for isolation of cardiac myocyte nuclei from a number of species including human, mouse, rat and rabbit [10••, 13•, 14••, 23••, 27]. Although PCM1 decorates adult cardiac myocyte nuclei in these studies, prenatal and early postnatal hearts seem to have low or variable PCM1 expression thus preventing isolation of nuclei with these markers at earlier stages of development [14••]. Thus, additional markers, including SIRPA (signal-regulatory protein alpha), have been applied to identify prenatal cardiac myocytes [28]. Recently, we have identified phospholamban (PLN) as a highly abundant and specific marker of cardiac myocyte nuclei in the mouse and human heart [14••, 24]. Using dual PCM1- and PLN-markers, cardiac myocyte nuclei could be isolated from human heart tissue with very high purity (≥ 98%) at fetal, infant and adult stages [14••]. Both markers resulted in a high degree of correlation of the nuclear transcriptomes obtained from adult hearts (*R*^2^ = 0.96) suggesting that PCM1 and PLN label the same population of cardiac myocyte nuclei [14••].

Using PCM1 and PLN as markers to isolate cardiac myocyte nuclei from human hearts by flow cytometry revealed changing cellular composition of the human heart between fetal and adult stages. The proportion of cardiac myocyte nuclei dropped from 75% in fetal ventricular tissue to 70% in infant hearts down to 30% in adult hearts [14••]. In terminally failing hearts, the percentage of cardiac myocyte nuclei was only 25% [14••]. In parallel, the percentage of diploid nuclei decreased from 75% in fetal cardiac myocytes to 30% in adult failing cardiac myocytes with higher abundance of highly polyploid nuclei (10% ≥ 16 n) in adult failing cardiac myocytes [14••]. Similar changes in cardiac myocyte nuclei composition were found during development and growth of the mouse heart, although mouse cardiac myocytes are mostly binuclear [13•, 29].

## Epigenetic Analysis of Cardiac Myocytes

Cardiac myocyte nuclei purified by flow cytometry from frozen left ventricular tissue were used for next generation sequencing of the transcriptome, DNA methylome (5mC, 5hmC) and seven histone marks as well as the chromatin structure in mouse and human hearts [14••, 24, 25]. To determine the human epigenome, cardiac myocyte nuclei were isolated from fetal (16–23 weeks of pregnancy), infant (1–12 months), adult non-failing and adult failing hearts [14••].

Results from nuclear RNA sequencing may profoundly differ from cellular RNA-seq experiments [23••]. In adult mouse cardiac myocyte nuclei, 60.8% of nuclear mRNAs were unspliced as compared with only 2.6% unspliced mRNAs in intact cardiac myocyte cells [23••]. Cellular to nuclear mRNA expression ratios differed by a factor of 2^16^ for all expressed genes, indicating a high degree of posttranscriptional alteration of transcript levels in cardiac myocytes [23••]. In total, 12,653 mRNAs were found to be expressed in adult mouse ventricular cardiac myocytes reflecting 63% of all coding genes in the mouse genome [23••]. Chromatin immunoprecipitation (ChIP-seq) of the histone mark H3K27ac identified 9187 active promoters in cardiac myocytes [23••]. Thus, expression of at least half of all coding genes can be detected in adult cardiac myocytes.

## Perinatal Adaptation and Physiological Growth

During physiological growth from fetal to adult stages, cardiac myocytes showed distinct features of transcriptome and epigenome changes. Whole genome bisulfite sequencing revealed a strong DNA demethylation at CpG sequences of promoters and gene bodies of cardiac myocyte genes. Gene bodies were demethylated in mouse and human cardiac myocytes and the level of DNA methylation correlated inversely with gene expression. Some of the highest expressed genes encoding for the sarcoplasmic ATPase SERCA (*Atp2a2*), the cardiac ryanodine receptor (*Ryr2*) or titin (*Ttn*) showed the strongest DNA demethylation (Fig. 1) [13•, 14••].

Interestingly, several genes which change their expression from prenatal to postnatal life also showed accompanying alterations in gene body CpG methylation [13•, 14••]. This was particularly apparent for genes encoding for sarcomere protein isoforms. Troponin I3 (*Tnni3*), which is not expressed prenatally but is strongly induced in postnatal cardiac myocytes, showed a gradual demethylation of its gene body until adulthood (Fig. 1) [13•]. In contrast, repression of the fetal troponin I1 isoform (*Tnni1*) was accompanied by de novo CpG methylation of its gene body between postnatal week 1 and adult life [13•]. A causal link between DNA methylation and gene expression could be demonstrated by ablation of expression of the de novo DNA methyltransferases 3A and 3B (DNMT3A, DNMT3B) in mouse cardiac myocytes in vivo. Knockout of DNMT3A/B prevented postnatal CpG methylation of the *Tnni1* gene body and partially rescued the repression of this gene [13•]. Overall, 440 cardiac myocyte genes switched the gene body methylation and expression during the perinatal period. This phenomenon was also observed in human cardiac myocytes in vivo [14••]. Perinatal isoform switches have been identified for several myocyte components including the sarcomere and mitochondria. The transition from skeletal (*Tnni1*) to cardiac troponin I (*Tnni3*) has been associated with changes in Ca^2+^ sensitivity of the sarcomere [30, 31]. Similarly, cardiac metabolism after birth rapidly switches to β-oxidation of fatty acids and involves expression of the adult isoforms of mitochondrial and other metabolic proteins [32]. Importantly, the final shape of the DNA methylome is formed continuously from fetal development to adulthood [14••] indicating that DNA methylation may not only be an important process during the initial phases of cardiac myocyte differentiation from progenitors but is also essential during cardiac myocyte switching and maturation after birth (Fig. 1).

## Cardiac Myocyte Epigenome in Chronic Heart Failure

In contrast to the 440 genes which change CpG methylation status of their gene bodies perinatally [13•], only 6 genes showed differential DNA methylation in adult failing cardiac myocytes without a consistent change in gene expression [14••]. Similar to DNA methylation, genome-wide chromatin compartments showed no or only subtle changes in mouse cardiac myocytes after pressure overload [19, 20]. While DNA methylation remained stable in heart failure, active histone marks H3K27ac and H3K36me3 were the best predictive marks for pathological gene expression in cardiac myocytes (Fig. 1). Together, these two marks explained 50% of the gene expression rank in failing human cardiac myocytes [14••]. In contrast to heart failure, myocardial ischemia activated a distinct gene expression and chromatin accessibility program [33••]. After experimental myocardial infarction in mice, border zone cardiac myocytes lost accessibility for regulatory elements containing the transcription factor MEF2, while injury-associated enhancers were more accessible for AP-1 binding sites [33••]. Thus, distinct injury types (pressure overload vs. ischemia) may induce separate transcriptome responses in adult cardiac myocytes.

## Gene Regulatory Regions in Ventricular Cardiac Myocytes

Analysis of genome-wide CpG methylation patterns identified short genomic stretches of low methylation (LMR, low methylated regions) which were primarily localized in intronic and intergenic regions and were characterized by high H3K4me1 and low H3K4me3 signals. Thus, these regions showed features of regulatory regions, including enhancers or repressors [14••]. Altogether, cardiac myocytes contained more than 100,000 LMRs, which were enriched for binding sites of cardiac transcription factors, including MEF2 (myocyte enhancer factor 2), GATA, CTF/NF1 and T-box [14••]. Similar to gene bodies, CpG methylation of LMRs was dynamic during physiological growth and disease of the heart [13•, 14••]. Eighteen percent of the LMRs were differentially methylated between fetal and adult cardiac myocytes. The majority of these regions showed a loss of DNA methylation until the adult stage which was accompanied by local accumulation of 5′-hydroxymethylcytosine (5hmC), thus reflecting the first step of active DNA demethylation by TET enzymes [14••].

In heart failure, 366 differentially methylated LMRs were identified as compared with > 18,000 LMRs with differential methylation during development. New LMRs that occurred in failing cardiac myocytes had a tendency for higher H3K27ac and H3K4me1 levels, but the next associated gene did not show a consistent change in expression [14••]. Cardiac myocyte LMRs were significantly enriched for single-nucleotide polymorphisms (SNPs) which have been associated with cardiac arrhythmia or coronary heart disease [14••]. Arrhythmia-associated LMRs showed typical features of cis-regulatory regions, i.e. enrichment of H3K4me1 and H3K27ac.

## Atrial vs. Ventricular Cardiac Myocytes

The heart consists of four chambers, two atria and two ventricles [34]. Because of their specialized functions, atrial cardiac myocytes differ from ventricular cardiac myocytes in various aspects. In terms of morphological features, ventricular cardiac myocytes possess broad transverse tubules while atrial cardiac myocytes show only few and short T-tubular structures [35]. Another difference between atrial and ventricular cardiac myocytes is the existence of atria-specific secretory granules as a sign for the specialized function in neurohormonal secretion [36]. Regarding electrical properties and action potentials, atrial cardiac myocytes show a less negative resting potential, a shorter duration as well as a more triangular shape of action potentials [37]. This is also evident in the specific expression of diverse channel subtypes and connexins [38]. Moreover, Ca^2+^ signalling during excitation-contraction coupling differs vastly between atrial and ventricular cardiac myocytes [39]. Previous analysis of the human myocardial transcriptome by microarray showed gene expression changes of 3300 and 2974 transcripts with higher expression in atria and ventricles, respectively [40]. In mouse tissue, gene expression profiling identified similar changes with 2099 ventricular genes and 2520 atrial genes [41]. Recently, Doll and colleagues identified many differences in proteomes of human atria and ventricles [42]. To gain insight into cardiac myocyte-specific gene expression differences, we isolated atrial and left ventricular cardiac myocytes and performed RNA sequencing [43••]. Overall, almost 400 genes were differentially expressed in atrial vs. ventricular cardiac myocytes (Fig. 1). Atrial cardiac myocytes showed differential expression of genes coding for structural proteins, ion channels, genes involved in energy metabolism or transcription factors compared with ventricular cardiac myocytes [43••]. The transcription factors hairy/enhancer-of-split related with YRPW motif 1 (*Hey1*), T-box 20 (*Tbx20*), T-box 5 (*Tbx5*) and the proto-oncogene AP-1 transcription factor subunit (*Fos*) showed increased expression in atrial cardiac myocytes, whereas Iroquois-related homeobox 4 (*Irx4*) was more highly expressed in ventricular cardiac myocytes [43••]. Thus, it will be essential to determine the molecular mechanisms that are involved in the development of the cellular and functional features of atrial and ventricular cardiac myocytes and to understand the plasticity in response to diverse stress signals.

## Transcription Factors Involved in Atrial Development and Disease

Several transcription factors including GATA, MEF2 and the homeobox transcription factor NKX2.5 have been shown to play an important role in cardiac remodelling and heart failure pathogenesis [44, 45]. Less is known about transcription factors and epigenetic programs that are involved in atrial remodelling and disease [46].

The chicken ovalbumin upstream promoter transcription factor II (COUP-TFII), also known as NR2F2 (nuclear receptor subfamily 2 group F member 2), has been shown to be important for atrial identity [41]. COUP-TFII belongs to the steroid thyroid hormone superfamily of nuclear receptors [47] and is involved in various processes like cardiovascular development, reproduction, neuronal development, organogenesis and metabolism [48]. COUP-TFII is highly expressed in atrial myocardium and only weakly expressed in ventricular myocardium [49]. Wu and colleagues showed that cardiac myocyte-specific knockout of COUP-TFII induces ventricularization of atria with ventricle-like electrical characteristics, increased cardiac myocyte size and development of T tubules [41]. Overexpression of COUP-TFII induces atrialization of ventricular cardiac myocytes. Therefore, COUP-TFII determines atrial identity during cardiac development through promoting atrial and suppressing ventricular gene expression. This included direct upregulation of atrial-enriched TFs *Tbx5* and *Hey1*, as well as downregulation of ventricular-restricted TFs *Hey2*, *Irx4* and *Lbh* [41].

Recent research has shown that TBX20, a member of the T-box transcription factor family plays also a key role in atrial development [50]. Tbx20 function in ventricular cardiac myocytes has been investigated earlier. Shen et al. deleted Tbx20 specifically in adult cardiac myocytes, which resulted in cardiomyopathy and arrhythmia [51]. While overexpression of Tbx20 in adult cardiac myocytes induced proliferation and improved cardiac function after myocardial infarction [52]. Besides the directly activating function of myocyte proliferation genes, Boogerd et al. showed that Tbx20 directly represses a cardiac progenitor gene program in cardiac myocytes and activates atrial and ventricular specific genes for the establishment or maintenance of atrial and ventricular identity [50]. Interestingly, at E10.5 and E11.5, atria from Tbx20 cKO mice showed reduced levels of COUP-TFII. Moreover, Boogerd et al. could show that Tbx20 binds an enhancer upstream of COUP-TFII, which regulates its expression in atrial cardiac myocytes while Tbx20 might establish ventricular identity by direct regulation of Hey2 and Irx4 in developing ventricular cardiac myocytes [50].

The cardiac T-box transcription factor Tbx5 was shown to regulate a network of genes to control atrial rhythm [53]. Tbx5 deletion in the adult mouse induced spontaneous and sustained atrial fibrillation with disruption of AF-susceptibility genes [53]. Moreover, Nadadur et al. demonstrated that Tbx5 and Pitx2 co-regulate a gene regulatory network essential for atrial rhythm [53]. A following study revealed the involvement of Tbx5-dependent non-coding RNAs that were generated from enhancers and correlated with target gene expression [54]). Subsequent investigations found that atrial arrhythmias caused by Tbx5 deletion can be rescued by reduced Gata4 levels while Nkx2.5 was dispensable [55].

## Transcription Factor ETV1 in Atrial Cardiac Myocytes

Recently, we identified the transcription factor ETV1 as an important component in the pathophysiology of atrial remodelling and atrial arrhythmia (Fig. 1) [43••]. ETV1 (E-twenty-six variant 1) belongs to the large family of ETS (E26 transformation-specific)-transcription factors which have a variety of functions. ETV1 was identified to play an important role in the development of the fast conduction system [56]. *ETV1* is highly expressed in murine pectinated atrial myocardium and the His-Purkinje system. Constitutive ETV1 knockout mice showed cardiac conduction defects and developmental abnormalities of the ventricular conduction system [56]. In line with this previous study, *ETV1* expression was significantly higher in atria than in ventricles. Furthermore, *ETV1* expression was significantly upregulated in atria from patients with permanent atrial fibrillation compared with sinus rhythm [43••]. To identify the potential role of ETV1 in atrial fibrillation, mice with cardiac myocyte-specific overexpression of ETV1 were generated. ETV1 overexpression induced atrial arrhythmia represented by loss of P-waves with various morphologies of QRS complexes. Atrial arrhythmia coincided with enlarged and dilated atria, interstitial fibrosis and atrial thrombus formation. Surprisingly, cardiac myocyte-specific expression of ETV1 did not influence ventricular morphology and function [43••].

In order to understand the mechanisms of ETV1-induced atrial remodelling precisely, a knockout mouse model was generated. Cardiac myocyte-specific ETV1-deficient mice (ETV1^MLCCre^) were generated by breeding mice carrying a floxed *ETV1* allele [57] with MLC2a-Cre mice [58]. ETV1-deficient mice were viable, and no differences in survival rate were observed between the genotypes. As angiotensin II (Ang II) activates signalling pathways that play a central role in the development of atrial remodelling and fibrillation [59], ETV1^MLCCre^ and control mice were treated with Ang II via osmotic pumps for 14 days. Interestingly, ETV1 ablation was protected from Ang II-induced atrial structural remodelling. RNA-seq analysis in atria was performed to determine the molecular basis of this protective effect. More than 1300 genes were differentially expressed by angiotensin II in control atria and showed no significant gene expression changes in ETV1^MLCCre^ atria. To identify ETV1 target genes specifically in atrial cardiac myocytes, we isolated cardiac myocyte nuclei from mouse atria by fluorescence-activated sorting. Chromatin accessibility in mouse atrial cardiac myocytes was analysed by ATAC-seq (assay for transposase-accessible chromatin sequencing). Moreover, chromatin immunoprecipitation for the active histone modification H3K27ac followed by high-throughput sequencing (ChIP-seq) was performed to identify active *cis-*regulatory regions in atrial cardiac myocytes. Nearly 10,000 regions were highly accessible and showed enrichment of H3K27ac in atrial cardiac myocytes. These regions were mainly found in promoter, intronic and intergenic regions. Moreover, ETV1 binding sites in atrial cardiac myocytes coincided with binding sites for other cardiac transcription factors, like TBX5, NKX2.5 and GATA4. Combining RNA-seq data with ETV1 binding motif containing active *cis*-regulatory regions resulted in the identification of 178 potential ETV1 target genes. Several have previously been associated with atrial arrhythmia or cardiac remodelling. Chromatin accessibility and gene expression analysis in mouse atrial cardiac myocytes strongly support that ETV1 orchestrates the regulation of a transcriptional network that drives atrial remodelling. These findings provide insights into the molecular mechanisms of atrial remodelling and arrhythmia [43••].

## Atrial Cardiac Myocyte Epigenome

Two very recently published papers provided information on chromatin accessibility in human atrial cardiac myocytes [60••, 61••]. Via combination of human transcriptomic, epigenomic and chromatin conformation datasets, van Ouwerkerk and colleagues showed a link between genetics and epigenetics in non-coding regions associated with atrial fibrillation [60••]. In the course of analysing long-range Pitx2c enhancer-promoter interactions involved in atrial fibrillation, Zhang and colleagues found enrichment for nearly 5000 ATAC peaks in left atrial cardiac myocytes compared with left ventricular cardiac myocytes [61••]. Further, cell-type-specific datasets are required to understand location and time-dependent mechanisms of epigenetic control to shape pathological gene expression during a heart disease.

## Conclusions

Cell-type-specific next generation sequencing techniques have provided detailed insight into epigenetic processes during differentiation, development, postnatal growth and disease of human and mouse cardiac myocytes. These studies revealed that the cardiac myocyte epigenome is shaped during cell specification and differentiation and remains highly dynamic until adulthood. Thus, the epigenome acquires different shapes in atrial vs. ventricular cardiac myocytes and during the timeline from embryonic development until adult life. Further studies are expected to fully unravel distinct epigenome features which separate atrial from ventricular myocytes in their physiological properties but also in their response to injury. Different cardiac diseases and signals may elicit distinct transcriptome responses in cardiac myocytes. Thus, transcriptomic changes induced by atrial arrhythmia differ profoundly from pressure overload, heart failure or myocardial ischemia. As highlighted by chamber-specific functions of the cardiac transcription factor ETV1 and the distinct chromatin features in pressure overload- vs. ischemia-induced injury, the responses to injury may greatly depend on the composition of the surrounding non-myocytes, location, time and disease signal influencing the cardiac myocytes (Fig. 1). Single-cell sequencing techniques are expected to add further cell-type-specific mechanisms and heterocellular interactions to the complex nature of heart disease. Future studies are required to fully unravel the four dimensions of the cardiac myocyte epigenome—atrial vs. ventricular location, time during development and growth, and disease-specific signals which may ultimately lead to new treatment strategies for heart disease.
